# Supplementary material for: The Gut Bacteria Dysbiosis Contributes to Chronic Graft-Versus-Host Disease Associated With a Treg/Th1 Ratio Imbalance
Source: Front Microbiol. 2022 Sep 8;13:813576. doi: 10.3389/fmicb.2022.813576 (PMC9493085; doi:10.3389/fmicb.2022.813576)
Supplement: Supplementary file 1 [file Data_Sheet_1.zip › P101SC18090073-01-B1-3-4_result/05.WebShow/krona/krona.html]

Javascript must be enabled to view this page.

members
count
unassigned
score
rank

MD1
MD2
MD3
MD4
MD5
MC1
MC2
MC3
MC4
MC5


70742707427074270742707427074270742707427074270742

14
domain
00000011

14
phylum
00000011

14
class
00000011

order
00000011
14

14
00000011
family

14
00000011
genus

70742707427074270742707427074270741707387074270742
domain
1111111111

0000000101
phylum
11

0000000101
class
11

0000000101
order
11

11
family
0000000101

genus
0000000101
11

2
phylum
000001

000001
class
2

000001
order
2

2
family
000001

genus
000001
2

1111111111
phylum
21181523192820192224

1111111111
class
21181523192820192224

21181523192820192224
order
1111111111

1111111111
family
21181523192820192224

21181323192820182224
1111111111
genus

00100001
genus
21

58965820294178227322558326
1111111111
phylum

58965820294178227322558326
1111111111
class

order
01000001
12

12
01000001
family

12
genus
01000001

58955820294178227322358326
1111111111
order

58955820294178227322358326
family
1111111111

58955820294178227322358326
1111111111
genus

phylum
1110111111
25121661990185013376

25121661990185013376
class
1110111111

25121661990185013376
1110111111
order

25121661990185013376
1110111111
family

25121661990185013376
1110111111
genus

2135585174143
phylum
111111111

class
111111111
2135585174143

2135585174143
order
111111111

2135585174143
111111111
family

genus
111111111
2135585174143

11828823973128614188281921003
phylum
1111111111

11828823973128614188281921003
class
1111111111

2
000001
order

000001
family
2

000001
genus
2

11828823973128613988281921003
order
1111111111

family
1111111111
11828823973128613988281921003

11828823973128613988281921003
1111111111
genus

1111111111
phylum
33772290142928915601325372010916980223544152028593

29233124522923365543
class
1111111111

29233124522923365543
order
1111111111

22172821432116304332
1111111111
family

genus
1111110111
678333264

1011111101
genus
22261245

1010111111
genus
22912444

12101313241612203019
1111111111
genus

genus
001110001
3313

family
1111111111
763398761211

14137
genus
0010010111

7623947594
genus
1111111111

class
1111111111
33158282432837414758307501882315561208864087027824

33158282432837414758307501882315561208864087027824
order
1111111111

1111111111
family
2653621458222289745234388833766090712199214150

5978466149761458508117081708151458912585
genus
1111111111

10010101
genus
2121

5129466
1111010001
genus

107579273771916236864643981733625
1111111111
genus

12581218122754412393811601621154740
1111111111
genus

1912101624204112210
genus
1111111111

168081346313728611814376570251616208116909556
1111111111
genus

genus
1111111111
6411210177396

1111111111
genus
48332910771219144138

17013519512187381727269
1111111111
genus

1798101165987
genus
1111110111

1111111111
genus
1150111912988518102551206991372508

155488138116
1111110111
family

genus
100100011
3333

741456383
1111110111
genus

51313723
1111110101
genus

1111111111
family
208280140165332203242203392508

1111111111
genus
208280140165332203242203392508

8191749615110382115110126
1111111111
family

415448496093644862108
genus
1111111111

32123
000110111
genus

genus
1111111111
13159443617321917

2722174046433261
1111110111
genus

000011111
family
64153

000011111
genus
64153

1111111111
family
758263232818353419355

1412131144273
genus
1111111011

617050222714313416652
genus
1111111111

6242632658644721678796497541114471816712979
family
1111111111

genus
1111111111
5395834872171611776479717296

1111111111
genus
91676681110361118

1111111111
genus
4413167141197

genus
1111111111
51423715941824910432

1111111111
genus
58453948595847756552

genus
1111111111
1782192619232034247931562841498844293030

2596256923041896251557034026561292808237
genus
1111111111

genus
1111111111
301252227214236697077331231

6181718141213422831
genus
1111111111

genus
1111111111
1931791659322179109148421233

1111111111
genus
3484383881432292872271421899642

354251253367057296226870170
1111111111
genus

13423
genus
111000011

111000011
family
11132

111000011
genus
11132

1111111111
class
1518215421317326122

1518215421317326122
1111111111
order

1518215421317326122
family
1111111111

1111111111
genus
1518215421317326122

3975205377001610512977912358547
1111111111
class

111111111
order
521114294

101001111
family
211281

genus
101001111
211281

family
110111011
3211313

3211313
genus
110111011

1111111111
order
3925185366991609508975903354547

family
1111111111
15220323835375019766129389214

1111111111
genus
15220323835375019766129389214

3888115151727
family
1111111111

3888115151727
genus
1111111111

237304290338848306299591263326
family
1111111111

237304290338848306299591263326
genus
1111111111

01000001
family
32

genus
01000001
32

class
1111111111
17321032611483732402488176157

17321032611483732402488176157
1111111111
order

17321032611483732402488176157
1111111111
family

1111111111
genus
99111236181751725731119

1001011011
genus
322132

311312
genus
101010111

1111111111
genus
6090808251207130439152136

genus
1100101111
5143431

387710621048
genus
1111111111

genus
0011001111
256311

30431335943336448312353993986845609434752739436336
1111111111
phylum

30431335943336448312353993986845609434752739436336
1111111111
class

5
order
00000001

00000001
family
5

5
genus
00000001

order
001
3

3
family
001

3
genus
001

order
1111111111
30431335923336148312353973986245605434672739436336

family
1111111111
308272278495375497666449380521

308272278495375497666449380521
genus
1111111111

family
1111111111
87413637872106623848271815737152027

31384124814623220
0001111111
genus

genus
1111111111
28685766812

1111111111
genus
25212635524126463126

1111111111
genus
84713347552060565416143813756531769

19830215722291323288212482617221291241231690818535
1111111111
family

19243209672239122874209622475621003235431659018296
1111111111
genus

5876055224142861416288580318239
1111111111
genus

918907724165897812561774217820001372
family
1111111111

918907724165897812561774217820001372
1111111111
genus

63413384647
1111111111
family

genus
1101101101
3153324

324881443
genus
1111011111

1111111111
family
8158774896

8158774896
genus
1111111111

245554105579845262572
1111111111
family

genus
1111111111
561810675891

genus
1111111111
19493695519140181671

1111111111
family
8463941985962063912106109761910315104735313796

8463941985962063912106109761910315104735313796
genus
1111111111

22641
01001111
order

22141
family
01001111

0000111
genus
214

genus
01000001
21

5
000001
family

genus
000001
5

order
00000001
2

00000001
family
2

2
genus
00000001

phylum
1111111111
20254119143838422447

6
001
class

order
001
6

6
family
001

6
001
genus

class
1111111111
19221819133837282447

101001001
order
1116

family
101001001
1116

1116
101001001
genus

1111111111
order
17181014123428251518

1111111111
family
17181014123428251518

genus
1111111111
17181014123428251518

order
001
4

001
family
4

001
genus
4

0001001
order
15

15
family
0001001

15
genus
0001001

14341343329
order
1111111111

1334343129
family
1111011111

1111011111
genus
1334343129

112
family
010010001

112
010010001
genus

1610
class
10100001

10100001
order
169

family
10100001
169

10100001
genus
169

1
order
00000001

family
00000001
1

genus
00000001
1

01001001
class
311

311
order
01001001

311
family
01001001

genus
01001001
311

class
00100011
1113

1113
order
00100011

1113
family
00100011

1113
00100011
genus

0010000101
phylum
1224

42
0010000001
class

42
order
0010000001

family
0010000001
42

genus
0010000001
42

class
0010000101
822

order
0010000101
822

5
family
001

genus
001
5

family
0010000101
322

genus
0010000101
322

1111111111
phylum
12282251364681524061

1111111111
class
12282251364681524061

81013244210263
1111111111
order

1111111111
family
8101324428263

genus
1111111111
8101324428263

00000001
family
2

2
00000001
genus

order
1111111111
418949324279421458

418949324279421458
1111111111
family

2
genus
0001

1111111111
genus
418947324279421458

6276741175725959238924466817442214334169
1111111111
phylum

5526691241694
class
1111111111

order
0110110111
3312441

3312441
0110110111
family

0110110111
genus
3312341

1
00000001
genus

1111111111
order
3264361621

51
family
0010001

51
genus
0010001

112
family
001011

112
genus
001011

32413621
1101110111
family

2313411
1001110111
genus

genus
1001
11

010000011
genus
221

000011
family
11

11
000011
genus

101111111
order
24254263

24254263
family
101111111

genus
000000011
23

genus
10111111
2425424

order
0000000001
2

2
family
0000000001

0000000001
genus
2

0010001
order
131

0010001
family
131

genus
0010001
31

10
001
genus

class
00001
3

3
00001
order

3
family
00001

3
genus
00001

556865736596538514142113590330778313444
class
1111111111

2
000001
order

2
family
000001

2
000001
genus

341061311339
1111111111
order

family
1111111111
341061311339

genus
1111111111
341061311339

47523338272924364129
1111111111
order

family
11011001
24123

genus
11011001
24123

45483337252924334129
1111111111
family

genus
1111111111
2411124443

genus
1110110101
3221313

112511
11111001
genus

genus
1111111111
1311710767548

1111111111
genus
8735436532

869555720
111111011
genus

10179116107101013
genus
1111111111

549164986535532913722057586730037763388
1111111111
order

family
1111111111
549164986535532913722057586730037763388

39924686471131028311178448213623831620
1111111111
genus

genus
1111111111
100711801305149529583112283501301573

genus
1111111111
491632518732246481571275263195

1116
10100001
genus

order
1111111111
211915105221019817

family
1111111111
146822851037

146822851037
1111111111
genus

1111111111
family
7137831459510

7137831459510
1111111111
genus

order
1011101111
63291631

11
10000001
family

00000001
genus
1

1
genus
1

53291531
family
1011101111

53261331
genus
1011101111

32
00001001
genus

7038339505689633219101329593721
class
1111111111

1111111111
order
7038339505689633219101329593721

family
1111111111
7038339505689633219101329593721

genus
110010111
166976

7008209485659553218991319587719
genus
1111111111

27232232
genus
1111101101

7126216118221123102
phylum
1111111111

1111111111
class
7126216118221123102

7126216118221123102
1111111111
order

7126216118221123102
family
1111111111

genus
1111111111
7126216118221123102

domain
0000000000
0000000000

0000000000
0000000000
phylum

0000000000
class
0000000000

0000000000
0000000000
order

0000000000
family
0000000000

0000000000
genus
0000000000
